# Probiotic potential of *Streptomyces* *levis* strain HFM-2 isolated from human gut and its antibiofilm properties against pathogenic bacteria

**DOI:** 10.1186/s12866-024-03353-x

**Published:** 2024-06-11

**Authors:** Jaya Verma, Sapna Devi, Anmol Narang, Sukhraj Kaur, Rajesh Kumari Manhas

**Affiliations:** Department of Microbiology, Guru Nanak Dev University, Amritsar, Punjab India

**Keywords:** Actinobacteria, Antibiotic resistance, Biofilm inhibition, Probiotics, *Streptomyces* spp.

## Abstract

**Background:**

Antimicrobial resistance (AMR) is a serious worldwide public health concern that needs immediate action. Probiotics could be a promising alternative for fighting antibiotic resistance, displaying beneficial effects to the host by combating diseases, improving growth, and stimulating the host immune responses against infection. This study was conducted to evaluate the probiotic, antibacterial, and antibiofilm potential of *Streptomyces levis* strain HFM-2 isolated from the healthy human gut.

**Results:**

In vitro antibacterial activity in the cell-free supernatant of *S. levis* strain HFM-2 was evaluated against different pathogens viz. *K. pneumoniae* sub sp. *pneumoniae, S. aureus, B. subtilis*, VRE, *S. typhi, S. epidermidis*, MRSA, *V. cholerae, M. smegmatis, E. coli, P. aeruginosa* and *E. aerogenes*. Further, the ethyl acetate extract from *S. levis* strain HFM-2 showed strong biofilm inhibition against *S. typhi, K. pneumoniae* sub sp. *pneumoniae, P. aeruginosa* and *E. coli.* Fluorescence microscopy was used to detect biofilm inhibition properties. MIC and MBC values of EtOAc extract were determined at 500 and 1000 µg/mL, respectively. Further, strain HFM-2 showed high tolerance in gastric juice, pancreatin, bile, and at low pH. It exhibited efficient adhesion properties, displaying auto-aggregation (97.0%), hydrophobicity (95.71%, 88.96%, and 81.15% for ethyl acetate, chloroform and xylene, respectively), and showed 89.75%, 86.53%, 83.06% and 76.13% co-aggregation with *S. typhi*, MRSA, *S. pyogenes* and *E. coli*, respectively after 60 min of incubation. The *S. levis* strain HFM-2 was susceptible to different antibiotics such as tetracycline, streptomycin, kanamycin, ciprofloxacin, erythromycin, linezolid, meropenem, amikacin, gentamycin, clindamycin, moxifloxacin and vancomycin, but resistant to ampicillin and penicillin G.

**Conclusion:**

The study shows that *S. levis* strain HFM-2 has significant probiotic properties such as good viability in bile, gastric juice, pancreatin environment, and at low pH; proficient adhesion properties, and antibiotic susceptibility. Further, the EtOAc extract of *Streptomyces levis* strain HFM-2 has a potent antibiofilm and antibacterial activity against antibacterial-resistant clinical pathogens.

**Supplementary Information:**

The online version contains supplementary material available at 10.1186/s12866-024-03353-x.

## Background

The emergence of multidrug-resistant pathogenic bacteria is a major concern in the healthcare system. They are responsible for the development of serious infectious diseases in humans, causing substantial health risks, especially in the aged and immunocompromised individuals, resulting in significant mortality and morbidity [1–3]. The life-threatening and most widespread antibiotic-resistant bacteria are methicillin-resistant *Staphylococcus aureus*, vancomycin-resistant *Enterococci*, and Extended Spectrum β-Lactamase (ESBL) producing *Pseudomonas aeruginosa, Klebsiella pneumoniae*, and *Escherichia coli* [4, 5]. These bacteria have been rapidly growing resistant to all the antibiotics currently available in the market, including macrolides, aminoglycosides, fluoroquinolones, and vancomycin [1].In addition, several bacterial pathogens form biofilms as a strategy to resist antibiotics, making them more indestructible than their corresponding planktonic forms [6]. It is estimated that biofilms are involved in over 60% of infections caused by microbes [7, 8] whereas two-thirds of all human bacterial infections are caused by biofilms [6]. These infections tend to be persistent as they resist antibiotics as well as immune defense mechanisms, and the treatment of biofilm infections causes a significant burden on the healthcare and medical sectors. Till date, there are no drugs that specifically target bacteria in biofilms; nevertheless, various approaches are in the early stages of development. Because of this reason, novel anti-biofilm agents with different targets and mechanisms of action are required [9, 10].

Actinobacteria are Gram-positive bacteria that comprise one of the largest phyla of bacteria and are widely distributed in both terrestrial and aquatic ecosystems. These bacteria are of great importance as producers of a plethora of bioactive secondary metabolites with wide clinical, aquaculture, veterinary and agricultural applications. They produce two-thirds of all the naturally produced antibiotics in current clinical use as well as numerous antibacterial, antifungal, antiviral and anticancer compounds [11].

In addition, biological control techniques including the use of probiotics have garnered growing attention as antibiotic alternatives during the last three decades. Probiotics are living microbes, when taken in adequate quantities may provide health benefits [12]. Probiotics have been recommended as promising alternatives to antibiotics for use in livestock production as prophylactic, therapeutic, and growth-promoting agents [13]. *Lactobacillus, Bifidobacterium, Bacillus, Enterococcus, Streptococcus* and *Candida* have all been utilized as probiotics in chickens and humans [14, 15]. However, to combat newly emerging communicable diseases and to improve health performance, novel probiotic strains are needed.

Though *Streptomyces*, one of the most interesting families of industrial bacteria, has been linked with pathogenicity and human infections [16], the study of streptomycetes present in the microbiome of healthy humans has been ignored, and their existence in human tissues is still underestimated [17]. The importance of *Streptomyces* in the human microbiome: healthy skin, gastrointestinal tract, respiratory tract, and uterus is reported rarely currently, and their associated probiotic features have yet only been established in some land vertebrate aquaculture [18–21]. However, the findings related to their biological properties recommend that they could be consumed by humans as probiotics [21]. Since the potential use of streptomycetes as probiotics in humans has not yet been studied, the functional properties of probiotic *Streptomyces* isolated from the human gut need thorough investigation.

With this view, the current work is focused on the evaluation of probiotic properties of strain HFM-2 such as viability in bile acid, gastric juice, pancreatin, and at low pH; proficient adhesion properties, and susceptibility to antibiotics. In addition to this, the EtOAc extract of *Streptomyces levis* strain HFM-2 has also been evaluated for antibacterial and antibiofilm activities against different pathogenic bacteria.

## Materials and methods

### *Streptomyces* isolate

*Streptomyces* isolate HFM-2 used in the present study was isolated from healthy human gut [22]. For the isolation of *Streptomyces*, stool samples were collected from healthy human hosts after taking their informed consent from the laboratory of Dr. Sukhraj Kaur, Department of Microbiology, G.N.D.U., Amritsar. The study was approved by the Institutional Human Ethics Committee, G.N.D.U.

### Test organisms

The human pathogens such as *Escherichia coli* (MTCC 1885), *Salmonella typhi* (MTCC 733), *Klebsiella pneumoniae* sub sp. *pneumoniae* (MTCC 109), *Pseudomonas aeruginosa* (MTCC 1688), *Streptococcus pyogenes* (MTCC 1927), *Staphylococcus epidermidis* (MTCC 435), *Staphylococcus aureus* (MTCC 96), *Enterobacter aerogenes* (MTCC 111), *Mycobacterium smegmatis* (MTCC 6), *Vibrio cholerae* (MTCC 3906) and *Bacillus subtilis* (MTCC 619) were procured from Microbial Type Culture Collection (MTCC) and Gene Bank, CSIR-Institute of Microbial Technology (IMTECH), Chandigarh, India. The clinical pathogens were methicillin resistant *Staphylococcus aureus* (MRSA) and vancomycin resistant enterococcus (VRE). Probiotic culture i.e. *Lactobacillus plantarum* (L14a and L14b) was procured from Dr. Sukhraj Kaur’s lab. All the bacterial cultures except *Lactobacillus* strains were maintained on nutrient agar slants in the refrigerator at 4 °C. *Lactobacillus* strain was cultured on De Man, Rogosa & Sharpe medium.

### Fermentation and extraction of antibacterial metabolites

The antibacterial metabolite production by *Streptomyceslevis* strain HFM-2 was carried out as described by Verma et al. [22]. The fermentation process was carried out in Erlenmeyer flasks on a rotary shaker at 180 rpm after the production medium was inoculated with *Streptomyces* culture. To prepare the seed culture, 7-day-old *Streptomyces* culture was inoculated in 100 mL SCNB (starch casein nitrate broth, pH 6). After 24 h, the inoculum (2%) was transferred aseptically into Erlenmeyer flasks (250 mL) containing the same medium (50 mL) and cultivated for five days at 28 °C at 180 rpm. Following fermentation, the culture broth was centrifuged at 10,000X g for 20 min at 4 °C to separate the cell-free supernatant. To extract the antibacterial metabolites, the cell-free supernatant was extracted twice with ethyl acetate in a 1:2 ratio (supernatant: ethyl acetate). The separated organic phase was treated with sodium sulfate (Na_2_SO_4_) to eliminate water content and then concentrated under vacuum using a rotavapor (BUCHI R-200) until completely dried.

### Stability of antibacterial metabolites in the cell-free supernatant of *S. levis strain* HFM-2

To investigate the thermostability of antibacterial metabolites produced by strain HFM-2, the cell-free supernatant was treated at various temperatures (-80 °C, -20 °C, 4 °C, 37 °C, 50 °C, 70 °C, 100 °C and 121 °C) for 1 h. In the same way, to assess photostability, the cell-free supernatant was exposed to UV and sunlights for one hour. The residual antibacterial activity of all the treated samples was determined against MRSA. Untreated supernatant was employed as a control.

### Antibacterial activity of *Streptomyces levis* strain HFM-2

The antibacterial activity was determined by a modified method of the Kirby Bauer antibiotic susceptibility test [23]. The Muller Hinton Agar (MHA) plates were seeded with 100 µL of test bacteria after setting their optical density (OD_600_) equivalent to McFarland standard [0.5] and 6 mm wells were made with sterile cork borer. After adding 100 µL of cell-free supernatant into each well, the plates were refrigerated for 1 h to allow active metabolites to diffuse, and then incubated at 37 °C. The results were expressed in terms of inhibition zones (mm) surrounding the wells after 24 h of incubation.

### MIC and MBC values of EtOAc extract of *S. levis* strain HFM-2

A microtiter plate (96-well) dilution experiment was performed to determine the MIC and MBC values of the EtOAc extract using the standard Kirby-Bauer disc diffusion method at different concentrations against *S. typhi, K. pneumoniae* sub sp. *pneumoniae, P. aeruginosa* and *E. coli.* Further, 100 µL of EtOAc extract and 100 µL of bacterial culture were prepared. The control blanks were filled with 100µL of EtOAc extract (different concentrations) along with 100 µL of nutrient broth. The positive control well was filled with 100 µL of bacterial culture and 100 µL of nutrient broth (NB), while the negative control was filled with 200 µL of NB simply. The plates were incubated for 24 h at 37 °C to obtain the OD. To determine MBC, nutrient agar plates were inoculated with higher concentrations of MIC broth that did not produce visible growth. Plates were incubated at 37 °C for 24 h. The minimum concentration with no bacterial growth was considered as MBC.

### The inhibitory effect of *S. levis* strain HFM-2 extract on biofilm-forming pathogenic bacteria

The inhibitory effect of EtOAc extract on the biofilm formation by different pathogenic bacteria was evaluated using a modified semi-quantitative plate assay [24]. For this 100 µL of bacterial suspension (OD_600_ = 0.5) was inoculated in 96 well flat bottom polystyrene plate containing 200 µL of nutrient broth. The plate was incubated for 48 h at 37ºC. Planktonic bacteria were removed by inverting the plate. Then 100 µL of EtOAc extract at various concentrations was added to the existing biofilm and the plate was incubated at 37ºC for 24 h. The plate was washed with sterile phosphate buffer (pH 7.4) saline and fixed with methanol at 65ºC for 1 h. Then 100 µL of 0.3% crystal violet was added to each well. After 5 min, the plate was washed with sterile double distilled water and dried. Subsequently, 200 µL of acetic acid was added to each well to dissolve the content in the well and OD at 595 nm was compared with the control. Biofilm which was not treated with extract served as control.

$$\eqalign{& {\rm{Percentage}}\,{\rm{of}}\,{\rm{biofilm}}\,{\rm{inhibition}}\,{\rm{ = }}\,{\rm{ }} \cr & {\rm{Absorbance}}\,{\rm{of}}\,{\rm{sample - }}\,{\rm{Absorbance}}\,{\rm{of}}\,{\rm{control}}\,{\rm{/}} \cr & {\rm{Absorbance}}\,{\rm{of}}\,{\rm{sample}}\, \times \,{\rm{100 \% }} \cr}$$

### Analysis of biofilm inhibition using fluorescence microscopy

To support the quantitative assessment of the biofilm formation, fluorescence imaging was performed with acridine orange staining technique [25]. The experimental setup consisted of 100 mm polystyrene petri dish with 50 mm glass slide; LB broth was used as an artificial nutritive-rich environment. A bacterial pathogen treated with and without extract was incubated at 37 °C for 48 h. Once after the luxurious development of biofilm on the slide, the slide was removed slowly, freed of planktonic cells by washing with PBS (PH 7.4), and fixed with 95% methanol for 30 s. Then, the slide washed with PBS, air dried, and stained with 0.1% acridine orange (1mL) for 10 min. The unbound stain was removed by washing with PBS, air dried, and observed under a fluorescence microscope (Olympus NX43).

### Antibiotic sensitivity test

The antibiotic sensitivity of *Streptomyces levis* strain HFM-2 culture was observed using Kirby Bauer disc diffusion method [23]. Different classes of clinical antibiotic discs were placed on SCNA plates seeded with *S. levis* culture and incubated at 28 °C for 7 days. After incubation, the zones of inhibition were measured in mm, and categorized as resistant (‘R’) and sensitive (‘S’).

### Survivability of *Streptomyces levis* strain HFM-2 culture

#### Preparation of *Streptomyces* culture inoculum

After inoculating starch casein nitrate broth (SCNB) with the *Streptomyces* isolate, the mixture was incubated for five days at 180 rpm at 28 °C. Following a 20-minute centrifugation at 10,000X g at 4 °C, the culture was recovered and subsequently washed three times using sterile PBS (pH 7.2). Using a UV spectrophotometer, the cells were re-suspended in the same buffer and adjusted to an OD_600_ of 1.0.

### Acid resistance test

To investigate the acid tolerance ability of *S. levis* strain HFM-2, a modified approach was used [26]. The strain HFM-2 (log_10_ CFU/mL) was re-suspended in sterile PBS adjusted to pH 2.0, 3.0 and 4.0 employing 1 N HCl (hydrochloric acid) and incubated at 28 °C for 180 min. Then 100 µL of aliquots were spread on SCNA plates and incubated for 7 days at 28 °C. Acid resistance was determined in triplicates in terms of viable colony counts.

### Gastric juice resistance assay

Tolerance of *S. levis* strain HFM-2 to gastric juice was determined with slight alterations as described by Maragkoudakis et al. [27]. *Streptomyces* culture suspension (log_10_ CFU/mL) was inoculated into sterile PBS (pH 1.5–3.5). The survivability of the isolate on SCNA medium was evaluated after 180 min of incubation at 28 °C.

### Bile resistance test

The bile resistance of the *S. levis* strain HFM-2 was assessed according to Hosseini et al., with slight alterations [26]. The *S. levis* strain HFM-2 culture (log_10_ CFU/mL) was suspended in sterile PBS (pH 7.8, 1 M NaOH) augmented with 0.3%,0.5% and 1% of oxgall (w/v). After 180 min of incubation at 28 °C, the viability of the culture inoculum was determined on the SCNA medium.

### Pancreatin resistance test

The pancreatin resistance of *S. levis* culture was tested according to the method described by Maragkoudakis after slight alterations [27]. *Streptomyces* suspension (log_10_ CFU/mL) was inoculated into sterile PBS (pH 7.0) augmented with pancreatin (1 mg/mL). Resistance was measured as viable colony counts using an SCNA plate after incubation for 180 min at 28 °C.

### Auto-aggregation test

Investigation of auto-aggregation was done as described by Agaliya et al. [28]. The aliquot of *S. levis treptomyces* (OD 1.0 at 600 nm) was taken in sterile test tube (4 mL) and incubated at 28 °C for 60 min. During subsequent incubation, the OD was measured at 600 nm. The auto-aggregation % was calculated using the following equation.

$${\rm{Auto - aggregation}}\,\left( {\rm{\% }} \right)\,{\rm{ = }}\,{\rm{ }}\left[ {\left( {{\rm{O}}{{\rm{D}}_{\rm{0}}}{\rm{ - O}}{{\rm{D}}_{{\rm{60}}}}} \right){\rm{/O}}{{\rm{D}}_{\rm{0}}}} \right]\,{\rm{*}}\,{\rm{ 100}}$$

where OD_0_ is the initial optical density, and OD_60_ is the optical density after 60 min of incubation.

### Cell surface hydrophobicity test

The BATH (bacterial adherence to hydrocarbons) technique was used to measure the degree of surface hydrophobicity. The method employed was as described by Sica et al. [29]. 4 mL of cell suspension (OD 1.0 at 600 nm) was added to 1 mL of each organic solvent, viz., chloroform, ethyl acetate and, xylene distinctly. The tubes were vortexed for 2 min to ensure thorough mixing, and then the mixture was set aside to stand for 60 min to ensure complete separation of the two phases. The water phase (aqueous) was separated and the OD was measured at 600 nm. A reduction in the OD of the water phase (aqueous) was used to quantify cell-surface hydrophobicity (H%), and the percentage of cells attached to the solvent phase (organic phase) was determined using the following formula, where ODb is the optical density of cell suspension before mixing and ODa is the optical density after mixing.

$${\rm{Hydrophobicity}}\,\left( {\rm{\% }} \right){\rm{ }}\,{\rm{ = }}\,{\rm{ }}\left[ {\left( {{\rm{1 - O}}{{\rm{D}}_{\rm{a}}}{\rm{/O}}{{\rm{D}}_{\rm{b}}}} \right)} \right]\,{\rm{*}}\,{\rm{100}}$$

### Co-aggregation test

The co-aggregation ability of *S. levis* strain HFM-2 with bacterial pathogens was assessed using a modified approach of Jankovic et al. [30]. In brief, in nutrient broth, the pathogens were grown at 37 °C for 24 h, and the cell suspension of each bacterial culture was made as described for *S. levis* culture. Then, an equal volumes (2 mL) of bacterial and *S. levis* strain HFM-2 suspensions (OD 1.0 at 600 nm) were combined in test tubes using a vortex. Control tubes had 2 mL suspension of each bacterium and strain HFM-2 culture. After 60 min of incubation, optical density was measured, and the percentage of co-aggregation was calculated using the following formula, where A represents absorbance, pro (probiotic), and p (pathogen) represents each of the two isolates in control tubes and pro + p represents their mixture.

$$\eqalign{{\rm{Co - }} & {\rm{aggregation}}\,\left( {\rm{\% }} \right){\rm{ }}\,{\rm{ = }}\,{\rm{[}}\left( {{{\rm{A}}_{{\rm{pro}}}}\,{\rm{ + }}\,{\rm{ }}{{\rm{A}}_{\rm{p}}}} \right){\rm{/}}\, \cr & {\rm{2 }}\,{\rm{ - }}\,{\rm{A}}\,\left( {{\rm{mixture}}} \right)\,{\rm{/}}\,{\rm{ }}\left( {{{\rm{A}}_{{\rm{pro}}}}\,{\rm{ + }}\,{\rm{ }}{{\rm{A}}_{\rm{p}}}} \right){\rm{/2}}\,{\rm{ *}}\,{\rm{ 100}} \cr}$$

### Haemolytic activity

The haemolytic activity of *S. levis* strain HFM-2 was investigated according to Karthik et al. [31]. The culture was inoculated on the blood agar plate and incubated at 37^°^C for 48 h. The plate was evaluated for haemolytic properties.

### Statistical Analysis

All the statistical analyses were performed in triplicates. Results were expressed in mean ± standard error (SE).

## Results

### Fermentation and recovery of antibacterial metabolites

The most effective solvent for recovering active metabolites from fermentation broth was found to be ethyl acetate. The isolated metabolites were concentrated using a rotary evaporator, resulting in an orange-colored dry extract that was redissolved in ethyl acetate.

### Antagonistic activity of *S. levis* strain HFM-2

**Fig. 1:**
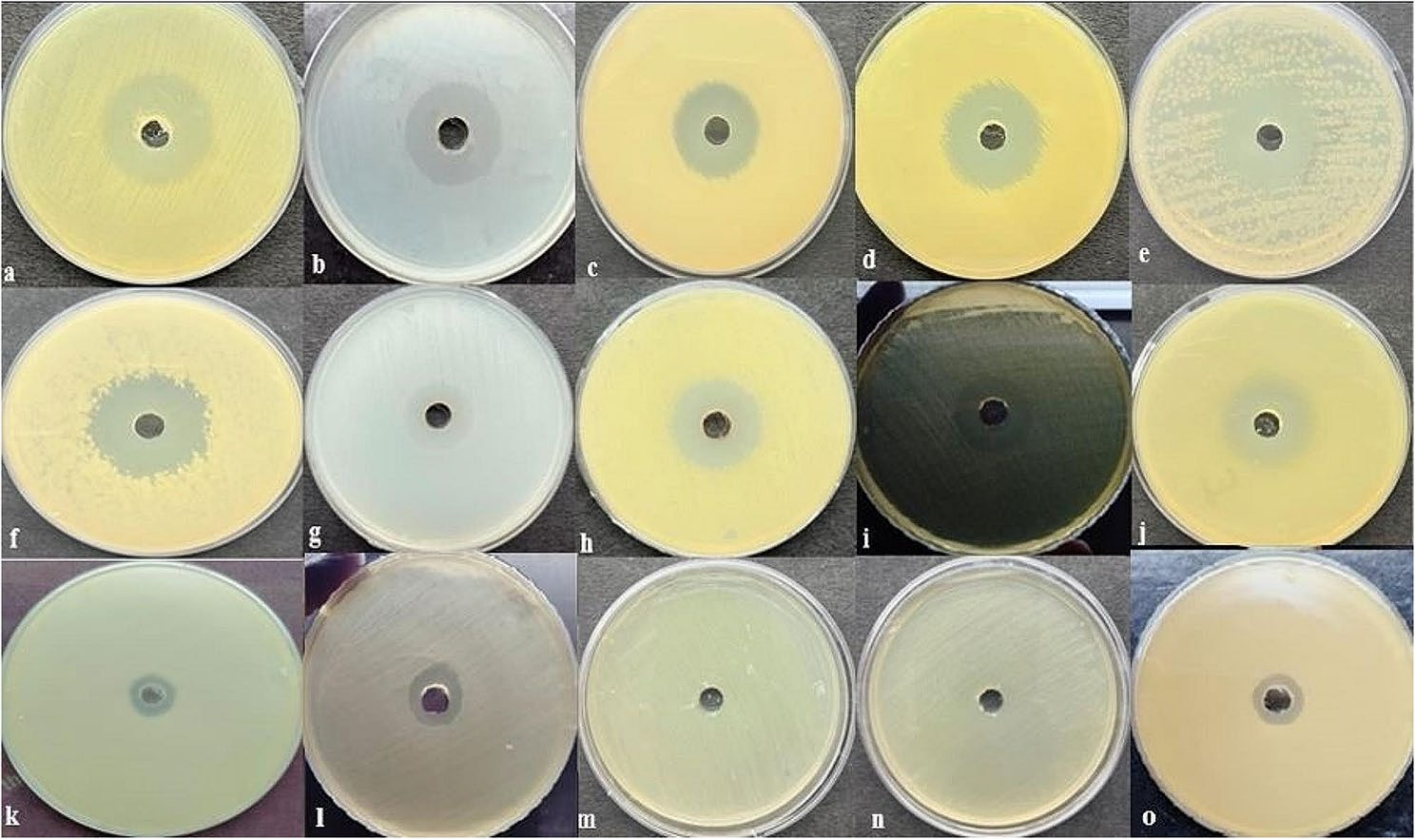
In vitro antibacterial activity of cell-free supernatant of *S. levis s*train HFM-2 by agar well diffusion method against different bacteria: (**a**) MRSA; (**b**) VRE; (**c**) *S. aureus*; (**d**) *K. pneumoniae* sub sp. *pneumoniae*; (**e**) *M. smegmatis*; (**f**) *S. typhi*; (**g**) *S. epidermidis*; (**h**) *B. subtilis*; (**i**) *V. cholerae* (**j**) *E. coli* (S1LF); k) *E. aerogenes*; l) *E. coli*; m) *L. plantarum* (L14a); n) *L. plantarum* (L14b); o) *P. aeruginosa*

In vitro bioassay confirmed the potent antibacterial activity of cell-free supernatant of *S. levis* strain HFM-2 against various tested bacteria. It demonstrated substantial suppression of pathogens such as drug-resistant MRSA, VRE, *E. coli* (S1LF), *S typhi, K. pneumoniae* sub sp. *pneumoniae, M. smegmatis* and *S. aureus*, with inhibition zones ranging between 25 and 28 mm. Moderate to weak activity was observed against *S. epidermidis, B. subtilis, V. cholerae*, *E. aerogenes, E. coli* and *P. aeruginosa* with 12–20 mm zones of inhibition, and no activity against probiotic strains *L. plantarum* strains (L14a and L14b) (Fig. 1 and Table S1).

### Determination of MIC and MBC values of EtOAc extract

MIC and MBC of the EtOAc extract were determined against various pathogens to evaluate the efficiency, and the nature of the activity whether it is bacteriostatic or bactericidal. *S. typhi* and *K. pneumoniae* sub sp. *pneumoniae* were found to be sensitive, with a MIC value of 500 µg/mL followed by *P. aeruginosa* and *E. coli.* MBC value of EtOAc extract was observed at 1000 µg/mL for *S. typhi, K. pneumoniae* sub sp. *pneumoniae*, *P. aeruginosa* and *E. coli*.

### Stability of antibacterial metabolites in the cell-free culture supernatant

Antibiotics should have a long shelf life, and also provide safe drug administration. From the perspective of commercialization, this property is also useful during the separation, purification, and processing of bioactive compounds. Antibacterial activity usually gets affected by extreme conditions. In the current study, antibacterial metabolites in the culture supernatant of *S. levis* strain HFM-2 were found to be thermostable up to 50 °C with a loss of only 7.15% after 1-hour treatment. However, a loss of 25% in residual activity was observed at 100 °C, and no activity was detected after autoclaving for 45 min. After 1 h of UV radiation and sunlight, a loss of 7.15% and 3.58%, respectively in antibacterial activity was observed (Table 1).

**Table 1:** Effect of various physical parameters on the antibacterial activity of culture supernatant of *S. levis* strain HFM-2

Treatment	*S. levis* strain HFM-2	
	Zone of inhibition (mm) against MRSA	% Residual activity
**Control (Untreated)**	28	100
**Heat Treatment**		
37^o^C, 1 h	27	96.42
50^o^C, 1 h	26	92.85
70^o^C, 1 h	25	89.28
100^o^C, 1 h	21	75
121^o^C, 45 min	00	00
**Low Temperature**		
-80^o^C, 1 h − 20 ^o^C 1 h 4 ^o^C 1 h	26 24 27	92.85 85.71 96.42
**Photostability**		
Sunlight, 1 h	27	96.42
UV light, 1 h	26	92.85

### Biofilm inhibition potential of *S. levis* strain HFM-2 extract against biofilm-forming pathogens

The EtOAc extract showed a concentration-dependent antibiofilm activity. The results of the assay showed inhibition of the biofilm with 81.07 ± 0.25% and 79.72 ± 0.33% at 250 µg/mL, 92.52 ± 0.33% and 91.47 ± 0.12% at 500 µg/mL against *P. aeruginosa* and *E. coli*, respectively. In the case of *S. typhi* and *K. pneumoniae* sub sp. *pneumoniae* biofilm inhibition of 76.1 ± 0.57% and 71.56 ± 0.25% at sub-MIC 250 µg/mL, and 93.02 ± 0.25% and 90.56 ± 0.57% at 500 µg/mL, respectively was observed.

### Analysis of Biofilm Inhibition using fluorescence microscopy

**Fig. 2:**
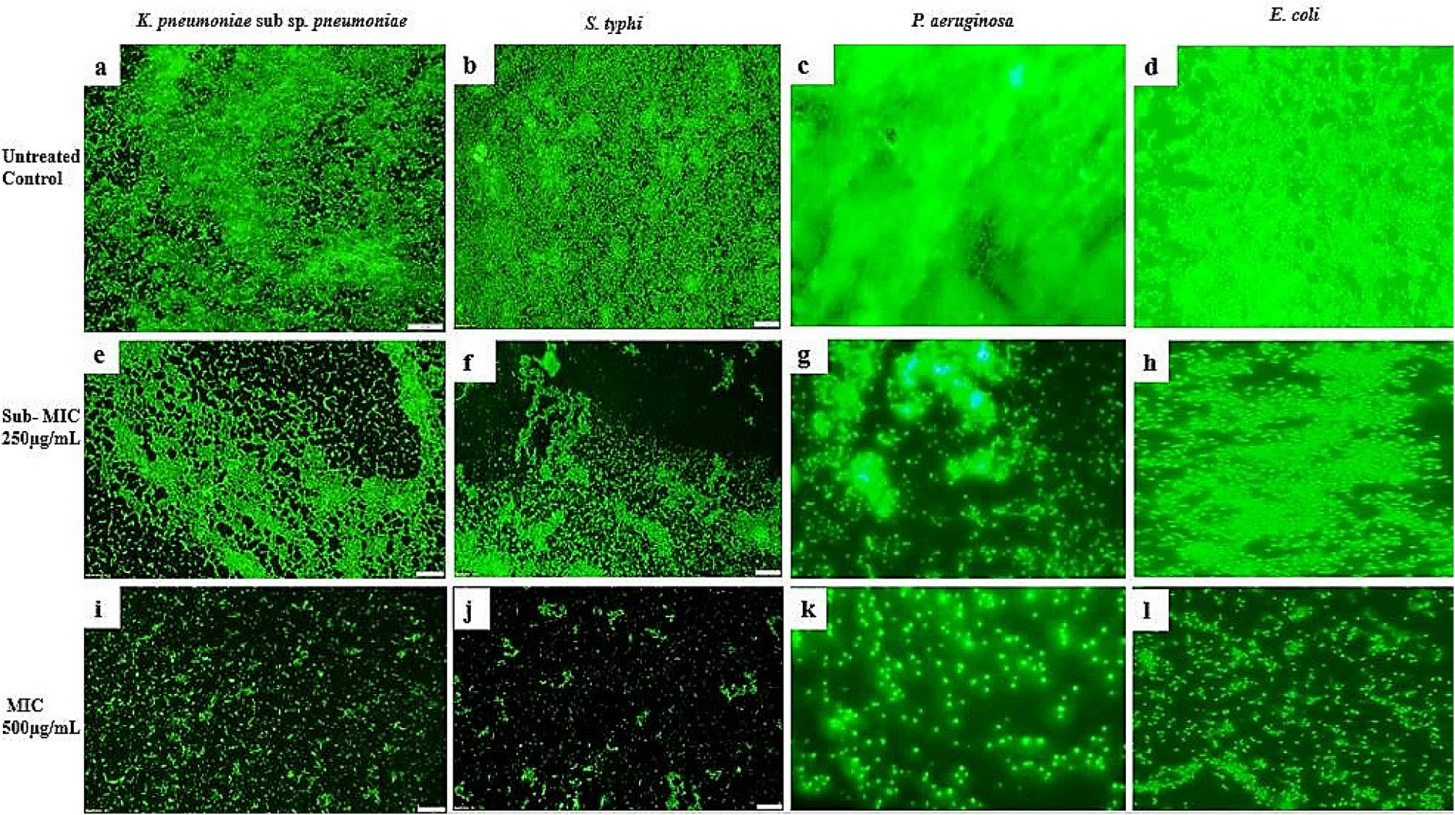
The fluorescence image of the effect of EtOAc extract of *S. levis* strain HFM-2 on different biofilm-forming pathogenic bacteria. **a-d**; untreated controls of *S. typhi; K. pneumoniae* sub sp. *pneumoniae*; *P. aeruginosa; E. coli*; **e-h**) at sub-MIC and i-l) MIC values

Further validation of the biofilm inhibitory activity of EtOAc extract was carried out by fluorescence microscopic studies after staining with fluorescent acridine orange dye. The fluorescence image of EtOAc extract treated and untreated pathogens showed a significant reduction in the biofilms of all the tested pathogens (*K. pneumoniae* sub-sp. *pneumoniae, S. typhi, P. aeruginosa*, and *E. coli*) at sub-MIC (250 µg/mL) and MIC (500 µg/mL) (Fig. 2).

### Antibiotic-susceptibility of *S. levis* strain HFM-2

The susceptibility of *S. levis* strain HFM-2 to various antibiotics was evaluated by Kirby Bauer disk diffusion assay and the zones of inhibition formed were determined (Fig. S1 and Table 2**).** Results showed that the strain HFM-2 was susceptible to all the verified antibiotics except penicillin-G and ampicillin.

**Table 2:** Antibiotic susceptibility of *S. levis* strain HFM-2

Antibiotic	Concentration (µg/unit)	Zone of inhibition (mm)	Susceptibility	
Streptomycin	10	40	S	
Gentamycin	10	35	S	
Kanamycin	10	41	S	
Ciprofloxacin	5	27	S	
Erythromycin	15	36	S	
Vancomycin	30	35	S	
Linezolid	30	42	S	
Clindamycin	2	11	S	
Meropenem	10	30	S	
Tetracycline	30	27	S	
Moxifloxacin	10	24	S	
Amikacin	30	30	S	
Penicillin G	2	-	R	
Ampicillin	10	**-**	R	

The experiment was carried out in triplicate. S-susceptible; R- resistant

### Survivability of *S. levis* strain HFM-2 culture

**Fig. 3:**
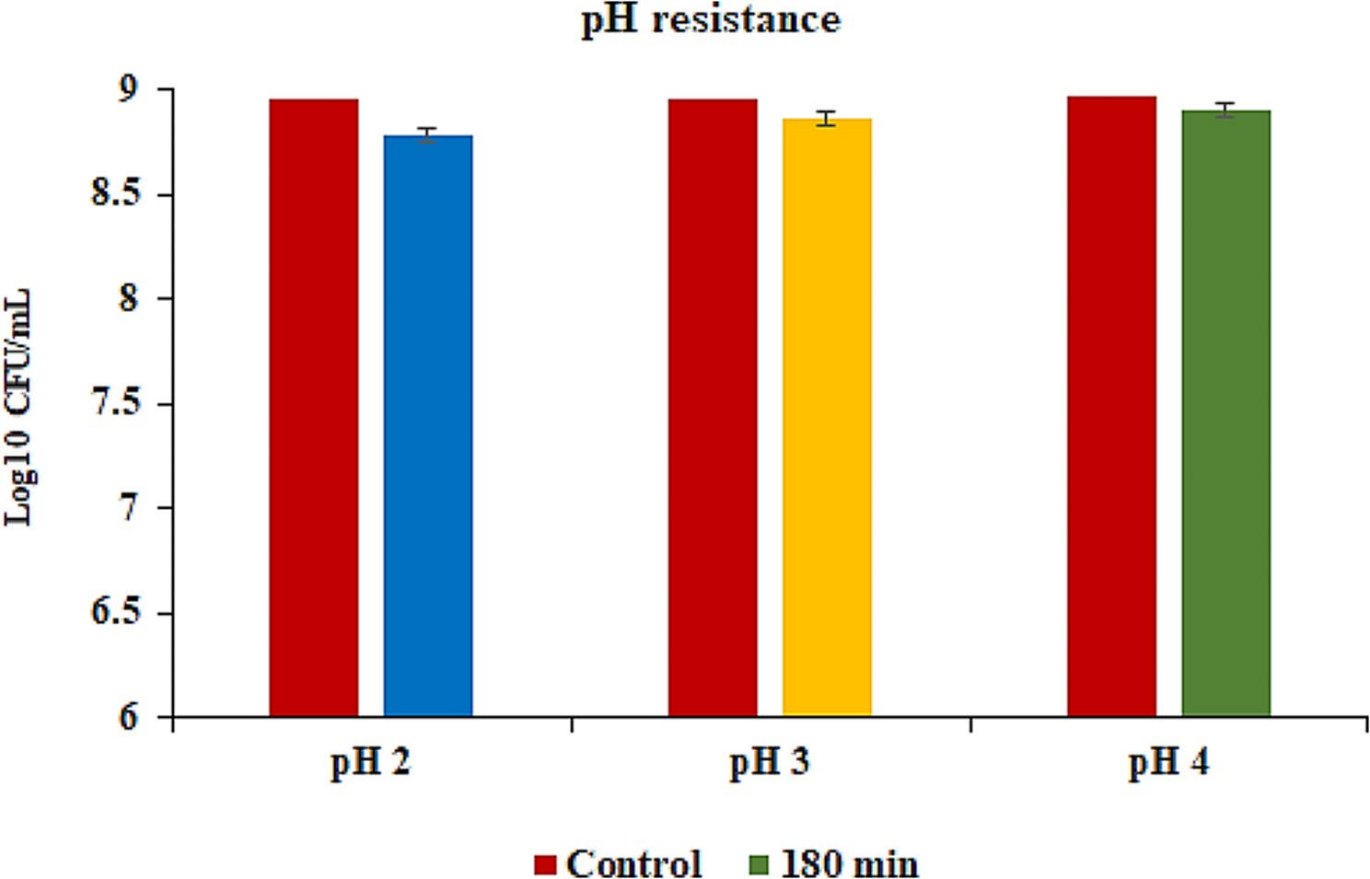
Survival of *S. levis* strain HFM-2 at different pH levels (2.0, 3.0, 4.0) after 180 min of incubation. Results are expressed as mean ± S.E of three replicate experiments

**Fig. 4:**
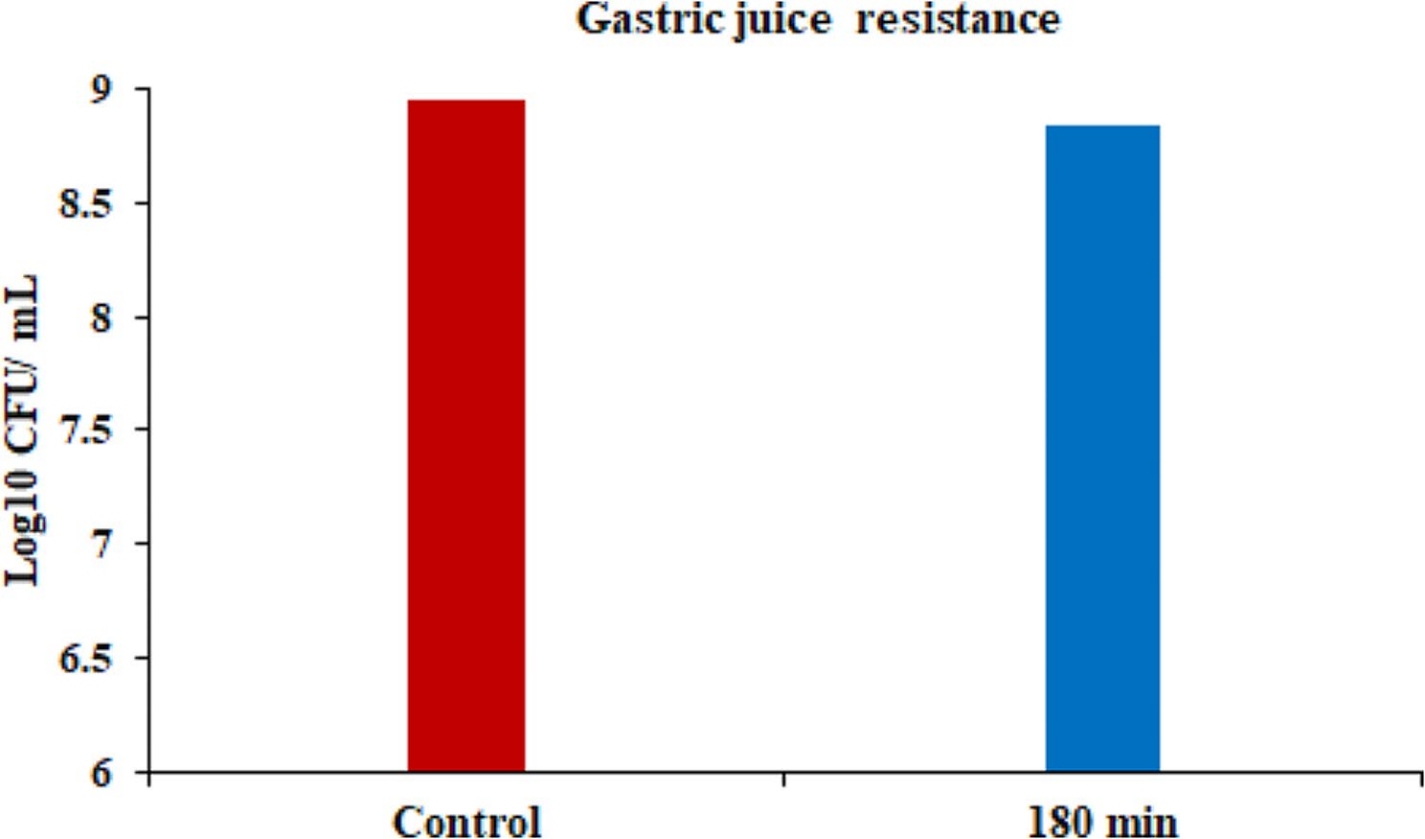
Survival of *S. levis* strain HFM-2 in the presence of gastric juice after 180 min of incubation. Results are expressed as mean ± S.E of three replicate experiments

**Fig. 5:**
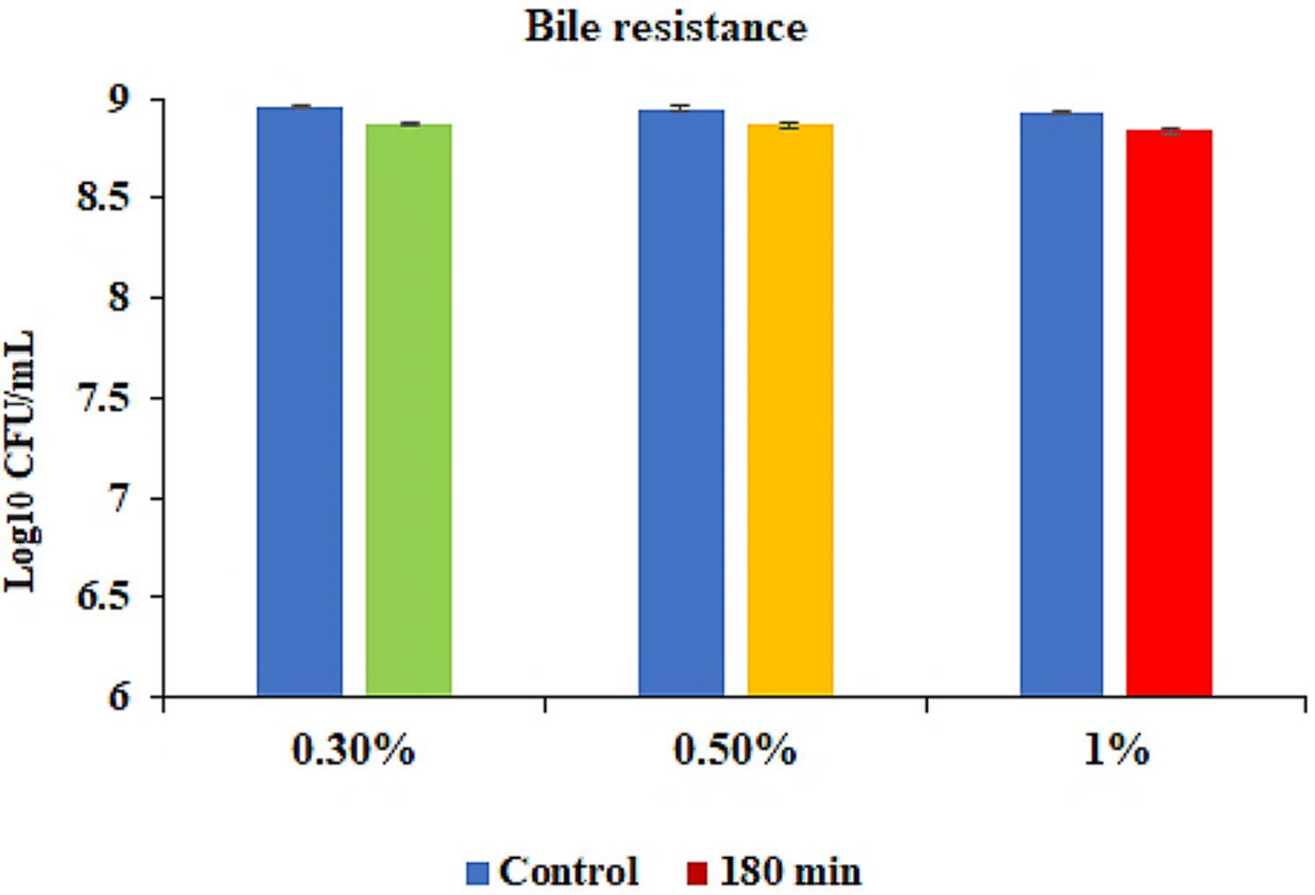
Survival of *S. levis* strain HFM-2 in the presence of bile at different concentrations after 180 min of incubation. Results are expressed as mean ± S.E of three replicate experiments

**Fig. 6:**
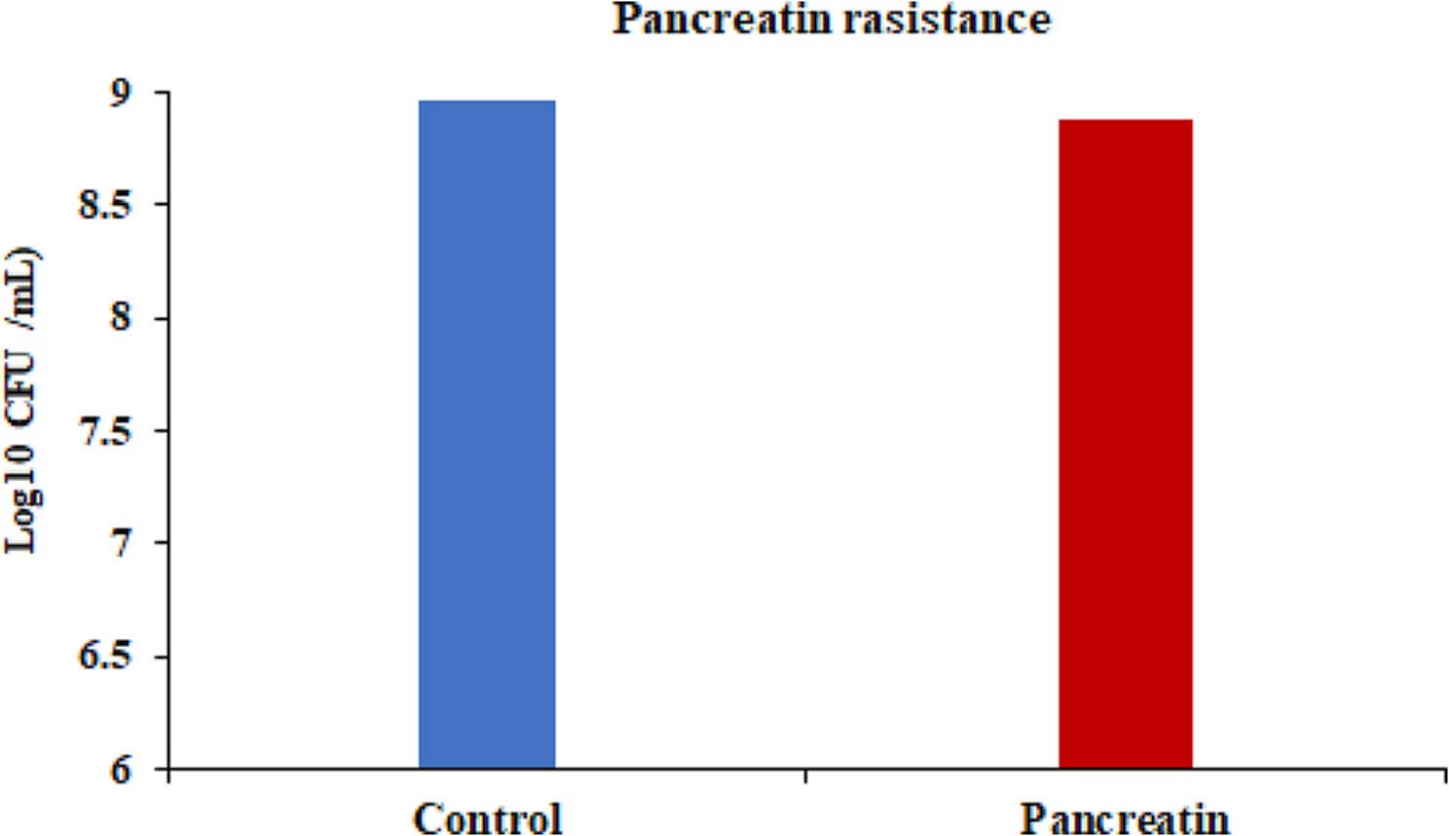
Survival of *S. levis* strainHFM-2 in the presence of pancreatin after 180 min of incubation. Results are expressed mean as ± S.E of three replicate experiments

The tolerance of *S. levis* strain HFM-2 to acid was determined at three different pH (2.0, 3.0, 4.0). In acid tolerance assay, survival rates of 8.78. 8.85, and 8.89 log_10_ CFUs/mL with minor log reductions of 0.17, 0.1, and 0.07 log_10_ CFUs/mL were observed after 180 min of incubation at pH 2.0, 3.0 and 4.0, respectively. So, the *S. levis* strain was able to survive in extremely acidic conditions (Fig. 3). Similarly, tolerance to gastric juice was determined after 180 min of incubation period in the presence of gastric juice. S. *levis* strain HFM-2 showed high levels of resistance to gastric juice with viable colony counts of 8.84 log_10_ CFUs/mL and a reduction of 0.11 log_10_ CFUs/mL (Fig. 4). The viability of *S. levis* strain HFM-2 with bile treatment was also determined after 180 min exposure. The minor log reductions of strain HFM-2 at 0.3, 0.5 and 1.0% of bile acid (Fig. 5) revealed its high resistance capacity to various concentrations of bile acid. Next to the bile acid resistance assay, the *S. levis* strain HFM-2 was tested for its resistance to pancreatin. The results revealed that the culture survived after 180 min of incubation with high survival counts of 8.87 log_10_ CFUs/mL and a reduction of 0.08 log_10_ CFUs/mL (Fig. 6).

### Cell surface hydrophobicity test

**Fig. 7:**
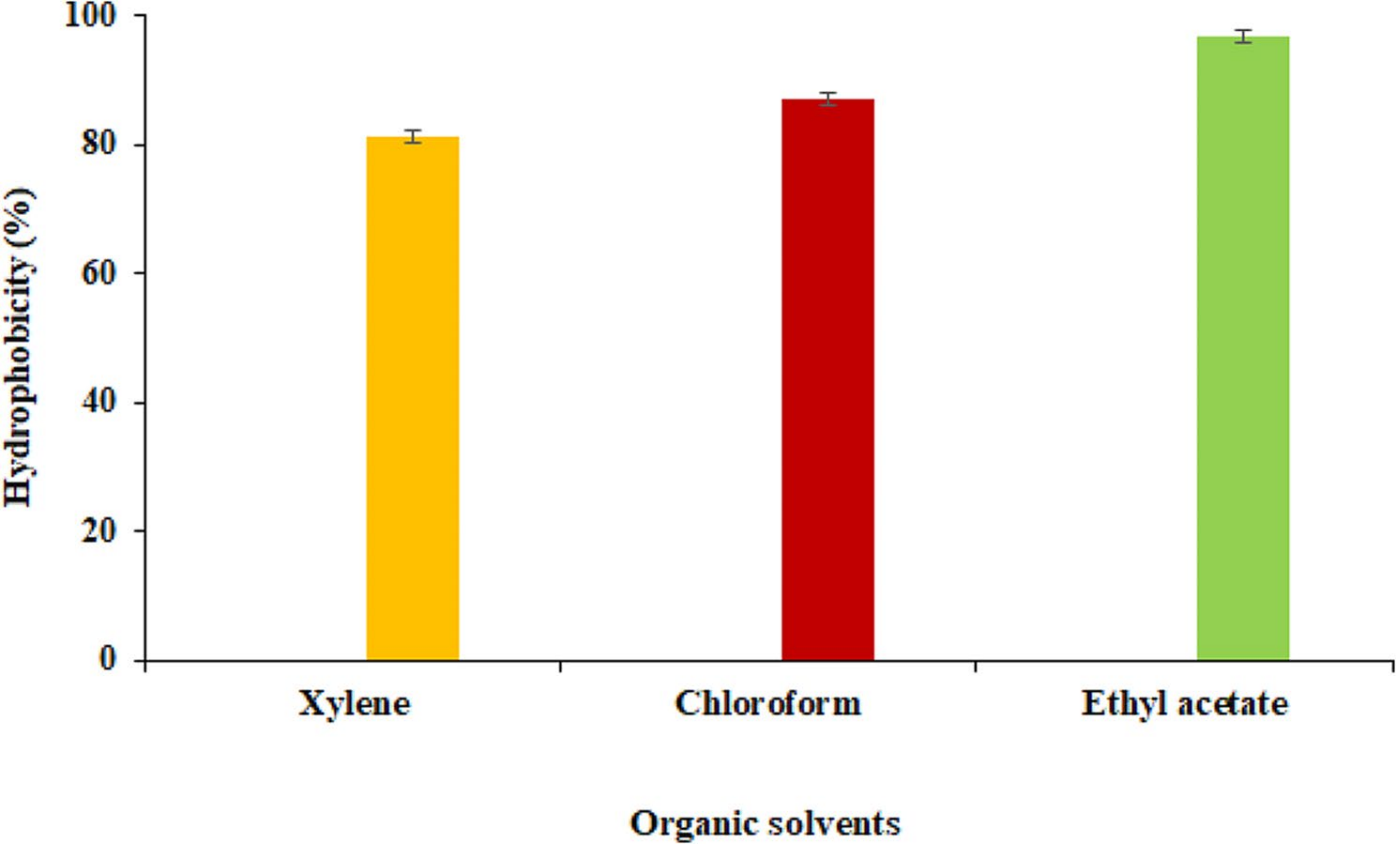
Cell surface hydrophobicity of *S. levis* strain HFM-2 with different hydrocarbons. Data represented as mean ± SD

The cell surface hydrophobicity of *S. levis* strain HFM-2 was measured based on its adhesion capacity for ethyl acetate, chloroform and xylene, the hydrophobic solvents. The strain HFM-2 was found to be strongly hydrophobic because of its high hydrophobicity values in the tested solvents. It showed adhesion capacity of 95.71%, 86.96% and 81.15% for ethyl acetate, chloroform and xylene, respectively after 60 min of incubation (Fig. 7).

### Colonization ability of *S. levis* strain HFM-2

**Fig. 8:**
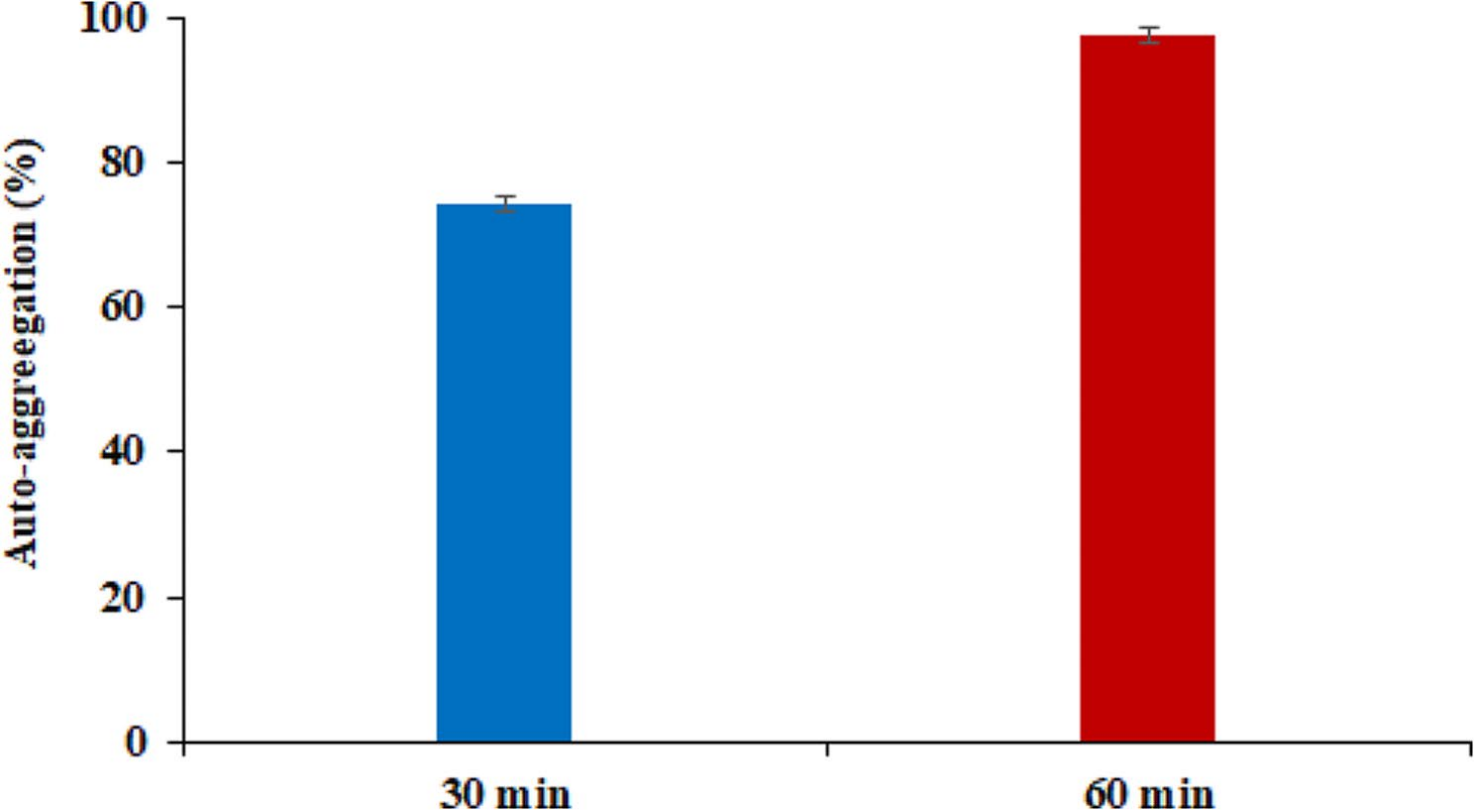
Adhesion properties of *S. levis* by auto-aggregation after 30 and 60 min of incubation. Data represented as mean ± S.E

The auto-aggregation ability of the *S. levis* strain HFM-2 was investigated based on its sedimentation characteristics. The *S. levis* strain HFM-2 displayed moderate to high auto-aggregation, with 74.3% and 97.36% at 30 min and 60 min of incubation, respectively (Fig. 8).

**Fig. 9:**
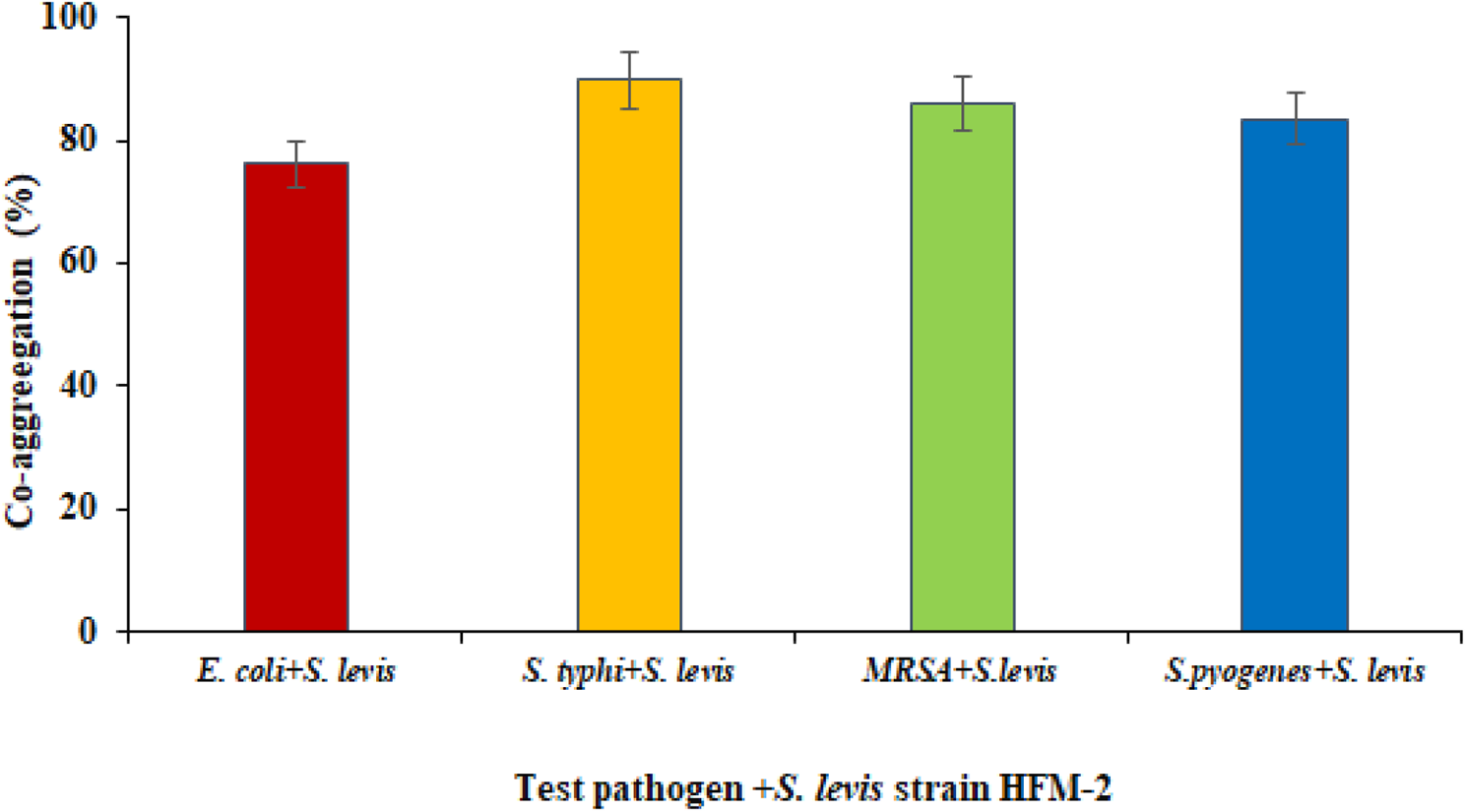
Co-aggregation ability of *S. levis* with various human pathogens. Results are expressed as mean as ± S.E of three replicate experiments

The co-aggregation assay is a reliable method to evaluate the close interaction between a probiotic and human pathogenic bacterium. In this study, the ability of the *S. levis* strain HFM-2 to co-aggregate with four different bacterial pathogens was evaluated. The strain HFM-2 showed high co-aggregation with *S. typhi* and MRSA (89.75% and 86.53%, respectively) and moderate co-aggregation with *S. pyogenes* and *E. coli* (83.06% and 76.13%, respectively) after 60 min of the incubation period (Fig. 9).

### Haemolytic activity

The haemolytic assay exhibited no haemolytic reaction of *S. levis* strain against human blood in the blood agar medium.

## Discussion

The rapid emergence of multidrug resistance among pathogenic microorganisms is posing a serious threat to the treatment of many infectious diseases as the drugs used to treat these diseases are ineffective and place an enormous cost on society [32].This resistance has reached a tipping point since there are limited options for treating some pathogenic bacteria, particularly those that cause hospital-acquired and community-acquired diseases [33]. Furthermore, biofilm-forming infections are becoming increasingly resistant to antibiotics, and troublesome in the clinical sector [34, 35]. Many bloodstream and urinary tract infections are associated with indwelling medical devices, which, in most cases, produce biofilms [36]. Surgical implants and medical technologies have significantly improved patients survival and rehabilitation from physical sickness [37]. However, they are perfect habitats for bacteria from patients skin, healthcare personnel skin, or in patient surroundings to colonize and create biofilms [38]. The increasing use of implanted medical devices, the possibility of biofilm formation on these devices, and the rise of drug-resistant strains have all imposed a significant burden on patients and healthcare systems [39, 40]. Therefore, there is a crucial and rising need to develop more effective drugs to fight antibiotic-resistant pathogens. Actinobacteria continue to be the most commercially and biotechnologically advantageous bacteria, producing 80% of the world’s antibiotics [41]. The metabolic capacity and genetic makeup of the *Streptomyces* genus provide a lot of potential as a source of biofilm-inhibiting metabolites. In the current study, the cell-free supernatant of the *S. levis* strain HFM-2 isolated from the human gut exhibited significant antibacterial activity against drug-resistant bacteria viz. clinical MRSA, VRE and *E. coli* (S1LF), *S typhi, S. aureus, K pneumoniae sub sp. pneumoniae, S. epidermidis, M. smegmatis, V. cholerae, E. aerogenes, B. subtilis* and probiotic *L. plantarum* stains (L14a and L14b). Rajan et al. reported the antibacterial activity of culture supernatant produced by *Streptomyces* sp. VITBRK2 isolated from marine deposit samples against drug-resistant vancomycin-resistant *Enterococci* (VRE) and methicillin-resistant *S. aureus* (MRSA) with 17 mm and 23 mm inhibition zones, respectively [33].

The MIC and MBC values of the EtOAc extract obtained from strain HFM-2 for different pathogenic bacteria were found to be 500 µg/mL and 1000 µg/mL, respectively. Tangjitjaroenkun showed a high MIC value i.e. 1 mg/mL of the ethyl acetate extract of *Streptomyces omiyaensis* SCH2 against *K. pneumoniae* [42]. Tangjitjaroenkun et al. reported 500–1000 µg/mL bactericidal activity (MBC) of *Streptomyces achromogenes* TCH4 extract against *S. aureus, S. aureus* (MRSA), and *K. pneumoniae* [43]. Similarly, Kurnianto et al. showed very high MIC and MBC values i.e. 2.50 mg/mL and 5 mg/mL, respectively of ethyl acetate extract of *Streptomyces* AIA12 against *Staphylococcus aureus* ATCC 259,232 and *Escherichia coli* ATCC 25,922 [44].

Additionally, the EtOAc extract of *S. levis* strain HFM-2 displayed biofilm inhibition against human pathogenic bacteria viz. *P. aeruginosa, E. coli, S. typhi* and *K. pneumoniae* sub sp. *pneumoniae* with the maximum of 92.52 ± 0.1, 91.47 ± 0.57, 93.2 ± 0.25 and 90.56 ± 0.57% inhibition, respectively at 500 µg/mL. Sumithra et al. and Goel et al. reported the antibiofilm activity of SeNPs (selenium nanoparticles) from *Streptomyces* sp. MA4 and AgNPs (silver nanoparticle) of *Streptomyces* sp. EMB24 against *P. aeruginosa* at 200 µg/mL and 50 µg/mL, respectively [41, 45, 48, 49]. Kim et al. showed antibiofilm activity of solvent extract from *Streptomyces* sp. BFI 230 against *P. aeruginosa* [46]. Similarly, Dhandapani et al. reported antibiofilm activity of active partially purified fraction isolated from *Streptomyces* sp. SRMA3 against drug-resistant clinical pathogens such as *E. coli* AMB4 (MK788230), *S. aureus* AMB6 and *P. aeruginosa* AMB5 [35].

Recently, Zhang et al. revealed the potential of the crude extract from *Streptomyces* strain to inhibit biofilm formation by *P. aeruginosa* with 53% inhibition at 5 mg/mL concentration which is very high as compared to HFM-2 extract [47]. Similarly, Chávez et al. displayed 50% biofilm reduction in the case of *K. pneumoniae* and *A. baumannii* by the culture supernatant from *Streptomyces pakalii* sp. [48]. According to the findings, the EtOAc extract derived from the *S. levis* strain HFM-2 exhibited higher inhibition against various biofilm-forming pathogenic bacteria in comparison to *Streptomyces* strains investigated in earlier studies.

Antibiotics should have a long shelf life, photo and thermo-stability, and provide safe drug administration. From the perspective of commercialization, this property is also useful during the separation, purification, and processing of bioactive compounds. In the current study, antibacterial metabolites in the culture supernatant of *S. levis* strain HFM-2 were found to be thermostable up to 50 °C for 1 h. However, a loss of 3.85%, and 25% in residual activity was observed at 70 °C and 100 °C, respectively, and no activity was detected after autoclaving for 45 min. After 1 h of UV radiation and sunlight exposure, a loss of 7.15 and 3.85%, respectively in antibacterial activity was observed. These reductions in antibacterial activity are due to the breakdown or structural changes of the active component under radiation. Hence, during production and storage, several conditions like light, temperature, etc. should be optimized to avoid the inactivation of metabolites [49].

Recent discoveries of positive qualities associated with actinobacterial metabolites have converted these microbes into potential probiotic candidates [50]. The advent of new infectious diseases necessitates the search for innovative probiotic strains to improve human health [51]. *Streptomyces* have been employed as probiotics since1940s, when *Streptomyces aureofaciens* probiotic was used to improve weight gain in animals, leading to the discovery of the antibiotic chlortetracycline [52]. Numerous studies show that *Streptomyces* has several beneficial impacts on aquaculture, including increased survival, feed conversion, growth rate, efficiency, and prevention of intestinal infections [53–56]. However, *Streptomyces* is less common in the human gut microbiome than in other non-human microbiomes [57]. The cause of decreased *Streptomyces* in human gut microbiota could be uncontrolled antibiotic usage [58]. As a result, *Streptomyces* probiotics provide a strategy for increasing these microorganisms in the human stomach to prevent diseases that are becoming more common as a result of our lifestyles [19]. To accomplish a probiotic status, microbes need to fulfill several criteria related to safety, and functional and technological properties.

The biosafety of probiotic microorganisms is a crucial aspect. Antibiotics used in food-producing animals are thought to stimulate the development of antibiotic resistance in the intestinal microflora, which can then be transferred to other harmful bacteria via genetic material exchange [59]. Thus, one of the safety concerns in probiotic research is the confirmation of microbial antibiotic-susceptibility patterns [60]. In our study, *S. levis* strain HFM-2 was found to be susceptible to all the tested antibiotics except ampicillin and penicillin-G. Some probiotic research conducted in recent years revealed that probiotic bacteria resistant to certain antibiotics might be useful for both preventative and therapeutic reasons in the treatment of intestinal infections. If given during and after antibiotic treatment, they can help to maintain or quickly restore the normal bacterial ratio in the intestines [61, 62].

The mucoid lining of the GI tract acts as a target for the exchange of various physiological substances [63]. Haemolytic activity might break down the epithelial layer of host cells, prompting the defense system. Failure of the defensive system might result in the host contracting invasive illnesses [64]. As a result, the absence of haemolytic activity throughout the screening technique is critical in determining whether the *S levis* strain HFM-2 is avirulent. The non-haemolytic nature of *S. levis* strain HFM-2 demonstrated that if it entered the food chain, it would not be fatal to the host and could be used as a probiotic for improved health and growth.

The probiotic microorganisms must be able to survive adverse host conditions such as low pH conditions of gastric juice (1.5–3.5) in the stomach, the action of pancreatic juice, and salivary enzymes [15, 65]. They must contribute to biological functions, such as controlling bacteria, removing toxins, and contributing to the host health, after effectively colonizing gut epithelial cells [66]. The acidic conditions in the gastrointestinal system operate as an efficient barrier against pathogenic microbe invasion and survival [67]. Therefore, tolerance to low pH is a key and crucial characteristic of probiotics.

The duration of food transit in the stomach is around three hours, depending on the kind of animal, feeding schedule, and development stage [68]. Particularly, when exposed to pH values of 2.5-4, *Lactobacillus* strains of food, human, and animal origin were able to maintain their survival [27]. However, Latha et al. reported that most of the *Lactobacillus* and *Enterococcus* probiotic strains survived better in the presence of pepsin at pH 3 rather than pH 2 [69]. According to Latha et al., several actinobacteria isolated from chicken were tolerant to pH 2 at high viability after 180 min of exposure [63]. In this study, *S. levis* strain HFM-2 was able to survive at pH 2.0, 3.0 and 4.0. Comparable results were reported for *Streptomyces* PDPF-2, which demonstrated high tolerance to acidic pH 2 [70].

In addition to low pH, the antimicrobial action of gastric juice provides very restrictive environments for the survival of intestinal microbes [71]. Therefore, the resistance of microbes to gastric juice is considered one of the most prerequisite properties of probiotics [63]. In this study, in the presence of gastric juice, *S. levis* strain HFM-2 showed high viable counts (8.84 log_10_ CFU/mL) after an exposure of 180 min of incubation, revealing good survivability. A recent study demonstrated a high tolerance of *S. flavotricini* isolated from grass carp to both acidic and alkaline conditions (pH between 2.0 and 11.0) [72]. Similarly, Latha et al. [63] revealed that *Streptomyces* isolates JD5, JD18, JD9 and JD11 isolated from chicken had moderate survivability in the presence of gastric juice.

The external environment of the small intestine, which includes pancreatin and bile, is the second important biological barrier [73]. They influence the survival of probiotics throughout their passage through the GI tract after bacteria have survived in the stomach barrier [74–76]. Therefore, the optimum probiotics for human or animal usage must be chosen based on the analysis of probiotic bacteria for resistance to bile and pancreatin [77]. Probiotic cultures often fare better in the simulated intestinal environment than in the stomach environment, according to the literature [78].

The detoxification process, which includes the deconjugation of bile salts, may have an impact on the ability of probiotic organisms in the GI tract to tolerate bile [79]. In this study, the *S. levis* HFM-2 exhibited high resistance to bile at 0.3% and showed minor log reduction at critical bile concentrations of 0.5% and 1% after 180 min exposure as compared to control. In addition, strain HFM-2 was also resistant towards pancreatin, with 8.87 log_10_ CFU/mL viable counts when compared to control.

Probiotic bacteria interact with mucus and epithelial cells of the small intestine, where they are easily removed by peristalsis. To provide long-term health benefits, probiotics must bind to the brush edge of the microvilli or the mucus layer of the GI tract [80]. Auto-aggregation and hydrophobicity are commonly utilized as markers of bacterial adhesion while they are directly connected to their capacity to adhere to the intestinal epithelium [15]. Latha et al. investigated the auto-aggregation ability of several *Streptomyces* strains isolated from chicken and found that isolate JD9 had the highest auto-aggregation (90.2%) followed by JD5 (86.9%) and JD4 (84.4%), with isolate JD15 having the lowest auto-aggregation of 12.4% [63]. In the present study, the *S. levis* strain displayed 97% auto-aggregation. The increased auto-aggregation capabilities of strain HFM-2 indicated that it would be highly helpful in forming biofilms and/or GI tract colonization processes that provide a barrier against colonization by pathogenic microbes.

Assessing microbial adhesion to hydrocarbons is a useful qualitative phenomenological method for estimating a bacterial strain’s adherence capacity [81, 82]. Latha et al. demonstrated the effective adhesion properties of the *Streptomyces* isolates, displaying hydrophobicity values of > 50%. The isolates exhibited different degrees of hydrophobicity, and the values ranged from 13.2 to 89.0%, 79.5–89.3%, and 27.3–88.1% for ethyl acetate, chloroform, and toluene, respectively [63]. Das et al. evaluated the probiotic potential of *Streptomyces antibioticus* EW1 and *Bacillus cereus* EW5 isolated from the digestive system of an earthworm *(Eisenia fetida*), as well as their probiotic effects on juvenile catfish (*Heteropneustes fossilis*). It was found to adhere with different organic solvents such as ethyl acetate, chloroform and xylene at 61.89%, 59.23% and 67.12%, respectively [56]. However, *S. levis* strain HFM-2 evaluated in this study exhibited strong hydrophobicity with adhesion capacity of 95.71%, 88.96% and 81.15% for ethyl acetate, chloroform and xylene, respectively indicating that it might have strong interactions with mucosal cells due to the occurrence of hydrophobic molecules on the surface.

The co-aggregation assay is a dependable approach for assessing the close contact of probiotic microorganisms with pathogenic bacteria [67]. This capacity might allow them to establish a barrier that inhibits pathogenic bacteria from colonizing them, and to release antimicrobial chemicals near pathogenic bacteria that inhibit their growth in the GI tract [61]. Latha et al. reported co-aggregation of *Streptomyces* isolates JD11 and JD18 isolated from chicken with different pathogenic bacteria, exhibiting 59.8% and 53.6% for *S. typhimurium* AP2; 64.5% and 53.9% for MRSA AP4; 51.9% and 53.5% for *E. coli* AP1; 59.1% and 56.9% for *P. multocida* AP3, respectively [63]. The strain HFM-2 evaluated in the present study showed variable degrees of co-aggregation with different clinical pathogens viz., *E. coli, S. typhi, S. pyogenes* and MRSA. The co-aggregation values exhibited were 76.13%, 89.75%, 83.06 and 86.53%, respectively after 60 min of the incubation. So, the *S. levis* strain HFM-2 has a high capability to co-aggregate with different tested clinical pathogens, suggesting that this property could allow it to survive at sufficiently high numbers and act as an efficient barrier to prevent colonization of intestine by pathogenic microorganisms.

## Conclusion

The study shows that the EtOAc extract of *Streptomyces levis* strain HFM-2 has a potent antibacterial and antibiofilm activity against antibacterial-resistant clinical pathogens. Further, *S. levis* strain HFM-2 exhibits significant probiotic properties such as good viability in bile, gastric juice and pancreatin, at low pH, proficient adhesion properties, and susceptibility to antibiotics. However, in vitro studies do not exhibit the significant potential of probiotics therefore, in vivo validations might be conducted further to evaluate the substantial behaviour of the selected probiotic.

### Electronic Supplementary Material

Below is the link to the electronic supplementary material.

Supplementary Material 1

## Data Availability

All data generated or analyzed during this study are included in this published article.

## Abbreviations

SCNA: Starch casein nitrate agar
MIC: Minimum inhibitory concentration
MTCC: Microbial type culture collection
PBS: Phosphate buffer saline
SCNB: Starch casein nitrate broth
NaOH: Sodium hydroxide
HCL: Hydrochloric acid
