# Supplementary material for: Probiotic potential of Streptomyces levis strain HFM-2 isolated from human gut and its antibiofilm properties against pathogenic bacteria
Source: BMC Microbiol. 2024 Jun 11;24:208. doi: 10.1186/s12866-024-03353-x (PMC11165917; doi:10.1186/s12866-024-03353-x)
Supplement: Supplementary file 1 — Supplementary Material 1 [file 12866_2024_3353_MOESM1_ESM.docx]

**Probiotic Potential of *Streptomyces levis* strain HFM-2 Isolated from Human Gut**

**and its Antibiofilm Properties against Pathogenic Bacteria**

**Jaya Verma, Sapna Devi, Anmol Narang, Sukhraj Kaur, Rajesh Kumari Manhas^*^**

**Department of Microbiology, Guru Nanak Dev University, Amritsar, Punjab, India**

***Corresponding author: Email: rkmanhas@rediffmail.com**

**Table S1: Antibacterial activity of cell-free supernatant from *S. levis* strain HFM-2 against various test bacteria**

| **Test bacteria** | **Zone of inhibition (mm)** |
| --- | --- |
|  | **Cell-free supernatant of HFM-2** |
| **Gram-positive bacteria** |  |
| ***B. subtilis*** | 21.0 ± 0.0.5 |
| ***S. epidermidis*** | 20.0 ± 0.25 |
| ***S. aureus***  ***M. smegmatis***  ***S. pyogenes*** | 25.0 ± 0.45  27.0 ± 0.5  19.0 ± 0.25 |
| **Gram-negative bacteria** |  |
| ***K. pneumoniae* sub sp. *pneumoniae***  ***E. aerogenes***  ***S. Typhi***  ***V. cholera***  ***E. coli***  ***P. aeruginosa*** | 22.0 ± 0.56  12.5 ± 0.56  26.0 ± 0.0  20.0 ± 0.0  14.0 ± 0.25  13.0 ± 0.0 |
| **Drug-resistant bacteria** |  |
| ***E. coli* (S1LF)** | 22.1 ± 0.5 |
| **Methicillin-resistant *S. aureus*** | 28.0 ± 0.25 |
| **Vancomycin-resistant *Enterococcus*** | 27.0 ± 0.35 |
| **Probiotic strains** |  |
| ***L. plantarum*** **L14a**  ***L. plantarum*** **L14b** | -  - |


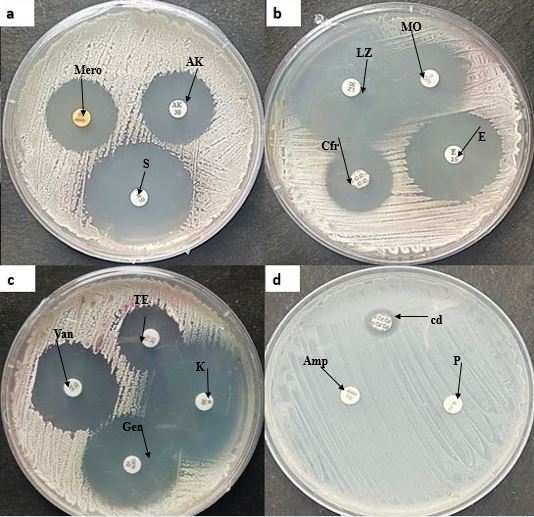


**Fig.S1: Antibiotic susceptibility test of *Streptomyces levis* strain HFM-2 with standard antibiotics a) Meropenum 30µg (Mero), Amikacin 30µg (Ak), Streptomycin 10µg (S); b) Linzolid 30µg (LZ), Moxifloxacin 10µg (MO), Ciprofloxacin 5µg (Cfr), Erythromycin 15µg (E); c) Tetracycline 30µg (TE), Vancomycin 30µg (Van), Kanamycin 10µg (K), Gentamycin 10µg (G); d) Clindamycin 2µg (Cd), Ampicillin 10µg (Amp), Penicillin G 2µg (P).**
